# Lack of evidence of viral reactivation in HBsAg-negative HBcAb-positive and HCV patients undergoing immunosuppressive therapy for psoriasis

**DOI:** 10.1186/s12876-014-0214-x

**Published:** 2014-12-19

**Authors:** Filomena Morisco, Maria Guarino, Serena La Bella, Luisa Di Costanzo, Nicola Caporaso, Fabio Ayala, Nicola Balato

**Affiliations:** Department of Clinical Medicine and Surgery, Gastroenterology Unit, University of Naples “Federico II”, Via S. Pansini, 5, Naples, 80131 Italy; Dermatology Units, University of Naples “Federico II”, Via S. Pansini, 5, Naples, 80131 Italy

**Keywords:** Biological drugs, HBV reactivation, HCV infection, Immunosuppressive therapy, Lamivudine, Psoriasis

## Abstract

**Background:**

HBV and HCV reactivation have been widely reported in patients undergoing immunosuppressive therapy (IT); however, few data are available on the risk of reactivation in patients with psoriasis receiving IT. The aim of our study was to assess the prevalence of HBV and HCV infection in patients with psoriasis and to evaluate the effects of IT during the course of the infection.

**Methods:**

The study included psoriatic patients who attended an Italian tertiary referral hospital from 2009 to 2012. A total of 224 patients were enrolled. We evaluated: HBV and HCV markers, type of IT and the occurrence of viral reactivation. The observational period ranged from the beginning of IT to the last visit, with a mean follow-up period of 54 months.

**Results:**

Two hundred and twenty patients (135 males and 89 females; mean age 59 years; range 18–86 years) with psoriasis, with or without psoriatic arthritis, receiving conventional IT and/or biological drugs were tested for markers of infection. We identified 23/224 patients (10.2%) with isolated positivity for HBcAb positivity, 36/224 (16%) with positivity for HBsAb/HBcAb, and 15/224 (6.6%) with positivity for HCV-Ab. No patient was HBsAg positive, none of them underwent pre-emptive therapy with lamivudine or other antiviral drugs and no one showed episodes of viral reactivation.

**Conclusions:**

The prevalence of HBsAg in patients with psoriasis is lower than that observed in the general population. The prevalence of isolated positivity for HBcAb and of combined positivity for HBcAb and HBsAb is 10.2% and 16%, respectively. The prevalence of HCV infection (HCV-RNA+) is 4%. In patients with psoriasis and HCV-Ab or HBcAb positivity, the IT seems to be safe, regardless of the type of drugs.

## Background

Psoriasis is a chronic, immune-mediated relapsing and remitting inflammatory skin and joint disease. The prevalence of psoriasis estimates as high as 2.8% in western populations [1,2]. Currently, different immunosuppressive therapeutic regimens are indicated for patients with psoriasis. The best treatment is determined on an individual basis and depends on the type of disease, the Psoriasis Area Severity Index (PASI) and comorbidities. For mild disease (PASI <10), involving only small areas of the body, topical treatments such as corticosteroids or calcineurin inhibitors or vitamin D derivates may be very effective and safe to use [3]. Up to 30% of 70% of psoriatic patients (PASI >/=10 that involves much larger areas of the body or for psoriatic artrhritis), require traditional systemic treatments such as retinoids, methotrexate and cyclosporine. Many of them imply long-term toxicity, treatment resistance and potential drug interactions; therefore, only 25% of psoriatic patients are completely satisfied with their treatment [4]. Advances in psoriasis therapies have introduced biologic agents, whose immune targeting is successful in treating many immunemediated inflammatory diseases [4]. Psoriatic patients who are refractory or intolerant to traditional therapy are the main candidates for biological anti-tumor necrosis factor alpha (TNF-a) drugs, for instance, infliximab, adalimumab, etanercept, golimumab or the anti-IL-12/23p40 monoclonal antibody, such as ustekinumab [5]. Several reports and studies have highlighted the risk of adverse events related to immunosuppressive therapy (IT) [4]. Under immunosuppression conditions, all patients with a history of exposure to HBV or HCV are at risk of reactivation [6-18]. The widespread use of biological drugs have raised these issues concerning the safety and the potential risks related to its administration, including patients with psoriasis [19-28]. Although a large amount of information on the relationship between IT for psoriasis and the behavior of HBV/HCV infections have become more available, the impact of different immunosuppressive drugs on the risk of reactivation remains poorly investigated.

The aim of our study was to assess the prevalence of HBV and HCV infection in a consecutive series of patients with psoriasis and to evaluate the effects of different schedules of immunosuppressive therapy during the course of the infection.

## Methods

This is a retrospective, observational study carried out at the Dermatology Unit of the University of Naples “Federico II,” a tertiary referral centre in Southern Italy. The target population consisted of adult patients with plaque-type psoriasis (Pso) with or without psoriatic arthritis (PsA) candidate to immunosuppressive therapy, observed from 1 January 2009 to 31 December 2012.

This study was independently designed by the authors, conducted in compliance with the 1975 Declaration of Helsinki and approved by the Ethic Committee of the University of Naples Federico II (protocol n° 175/2012).

Records for 224 outpatients were reviewed in relation to the markers of previous disease or active HBV and HCV infection. Among them, patients with almost 1 positive marker of HBV or HCV infection were identified for the inclusion in the study. All of the selected patients underwent immunosuppressive therapy, such as conventional immunosuppressive treatment (cyclosporine A, methotrexate (MTX)) or biological treatment (adalimumab, etanercept, infliximab, golimumab, ustekinumab) or combined biological plus methotrexate. The medical records of these selected patients were retrospectively reviewed.

Before starting the immunosuppressive therapy, all psoriatic patients had been routinely tested for serology of HBV and HCV infection. For patients with HBV or HCV infection, a scheduled monitoring had been applied: transaminases every 1 month, complete liver function test (bilirubin, INR, γ-Glutamyltransferase, alkaline phosphatase, albumin) every 3 months and HBV/HCV serological status (HBsAg and HBV-DNA for HBV infection, HCV-RNA for HCV infection) every 3 months from the start of therapy, until the last follow-up visit.

### Definition of HBV and HCV infection

HBV chronic infection was defined according to the EASL guidelines [29] as patients positive for HBsAg, independently from the HBeAg/HBeAb positivity and the levels of HBV-DNA. “Occult” HBV infection (OBI) represents a particular clinical entity that is characterized by the persistence of HBV DNA in the liver tissue, without the evidence of overt HBV infection in individuals who are HBsAg negative and HBcAb positive either with or without serum HBV-DNA positivity [29]. The difficulty in identifying HBV-DNA in liver biopsy (frequently not justified in patients without clinical signs of hepatitis) and the rarity of detectable serum viremia, even with sensitive techniques, lead to consider all HBcAb positive patients (HBsAg and HBV-DNA negative, with or without HBsAb positivity) as potential OBI. Patients with HBsAg negativity but positivity for HBcAb with HBsAb positivity, were considered as a resolved HBV infection [29].

Potential OBI and resolved HBV infection were analyzed for the same parameters as the HBsAg-positive patients. HCV infection was defined as patients positive for anti-HCV, with or without positivity for HCV-RNA [30].

### Definition of HBV and HCV reactivation

In HBV- positive patients, the following biochemical events were considered significant for a viral reactivation:

- in HBsAg-positive patients (active or inactive carriers), the increase of at least one logarithm of HBV-DNA with or without the concomitant increase of transaminases;
- in potential OBI and resolved HBV infection (HBsAb positive and/or HBcAb positive patients), the re-emergence of HBsAg or the appearance or increase of at least one logarithm of HBV-DNA.

Reactivation of HCV was defined as a significant increase of HCV-RNA (at least one logarithm), with or without a concomitant increase of transaminases. In HCV-Ab positive but HCV-RNA negative patients, the re-appearance of HCV-RNA was considered as a relevant event.

### Serological profile

HBsAg, HBcAb and HBsAb were determined by conventional commercial assay kits (Abbott AxSYM AUSAB, Germany; HBsAg EIA, Abbott, North Chicago, IL). HCV-Ab and HDV-Ab were determined by commercial enzyme linked immunosorbent assay III, Abbot Laboratories Chicago. All HBcAb-positive samples were assayed for serum HBV-DNA by a commercial qualitative target amplification method (Cobas Ampliscreen, Roche Molecular Systems, Branchburg, New Jersey, USA). In order to achieve the highest sensitivity allowed by this method (20 IU/mL), testing was performed on each individual sample without pooling and increasing the volume for extraction (500 μL). The specimens that resulted positive were further tested by a quantitative method (Cobas Amplicor HBV Monitor, Roche Molecular Systems, Branchburg, NJ, USA) to determine the viral load.

All HCV-Ab positive samples were assayed for serum HCV-RNA by a qualitative method (Cobas Ampliscreen, Roche Molecular Systems, Branchburg, New Jersey, USA). In order to achieve the highest sensitivity allowed by this method (15 IU/mL), testing was performed on each individual sample - without pooling- and increasing the volume for extraction (500 μL). The specimens that resulted positive were further tested by quantitative method (Light Cycler Instrument, Roche Molecular Biochemicals, Mannheim, Germany) to determine the viral load.

### Statistical analysis

Demographical, clinical, biochemical, histological, virological and therapeutic data were collected from medical records by case report forms. Baseline characteristics were expressed as median and range for continuous and not normally distributed data, as a mean and standard deviation (SD) for normally distributed data and as a percentage for categorical data.

## Results

Seventy-four patients with plaque-type psoriasis (Pso) with or without psoriatic arthritis (Psa) and with active or previous HBV or HCV infection were identified in a population of 224 patients, receiving conventional or biological treatment. All of these patients are still being treated for psoriasis. The characteristics of our population are illustrated in Table 1.

**Table 1:** **Characteristics of patients with psoriasis at baseline**

	**Overall**	**Isolated HBcAb positive**	**HBcAb/HBsAb positive**	**HCV-Ab positive**
**N° of patients**	224	23	36	15
**Age, yrs (mean ± SD)**	49 [±13.3]	66 [±10.6]	52 [±12.4]	62 [±11.8]
**Gender (M/F) N°%**	135/89 [60.2/39.7%]	10/13 [43.4/56.5%]	27/9 [75/25%]	11/4 [73.3/26.6%]
**Pso**	113 [50.5%]	10 [43.5%]	22 [61.1%]	9 [60%]
**PsA**	111 [49.5%]	13 [56.5%]	14 [38.9%]	6 [40%]
**ALT, U/L (mean ± SD)**	23 [±5.3]	27 [±2.3]	24 [±3.2]	25 [±2.1]
**HBV-DNA positivity**	0/224	0/23	0/36	0/15
**HCV-RNA positivity**	9/224	0/23	0/36	9/15
**HBsAg positivity**	0/224	0/23	0/36	0/15

### HBV infection

No patient was HBsAg positive. Globally, 59 patients showed almost 1 marker of HBV infection; 23/224 (10.2%) patients were isolated HBcAb positive (OBI) and 36/224 (16%) patients were HBsAb and HBcAb positive (resolved HBV infection). The main features of the 59 patients are reported in Table 1. Thirty-two patients were affected by plaque-type psoriasis and 27 by psoriatic arthritis. None of them showed signs of cirrhosis. At baseline all patients had normal aminotransferase levels and negative HBV-DNA. All HBV patients underwent immunosuppressive therapy. The treatment schedules are reported in Table 2. Nineteen patients (32%) were treated with conventional drugs only (Metotrexate, Cyclosporine or a combination of the two drugs) for a mean time of 24 months, 34 patients (58%) were treated with conventional and biological drugs, sequentially, for a mean time of 42 months, and 6 patients (10%) were treated with conventional drugs followed by a combination of biological drug plus methotrexate) for a mean time of 72 months. No patients were treated with prophylactic Lamivudine. In all of the cases serum liver functional tests showed no significant changes in AST or ALT levels and HBV-DNA positivity, from baseline to the last follow-up visit.

**Table 2:** **Schedules of treatment in patients with HBV or HCV infection**

**Schedules of treatment [#]**	**Isolated HBcAb positive (23 patients)**	**HBcAb/HBsAb positive (36 patients)**	**HCV-Ab positive (15 patients)**
**Conventional therapy**			
MTX, n.	**3**	**5**	**0**
*Duration, months (mean ± SD)*	11 ± 7,54	24 ± 23,62	-
Cyclosporine, n.	**0**	**4**	**4**
*Duration, months (mean ± SD)*	-	5,75 ± 4,5	20,25 ± 10,34
MTX + Cyclosporine, n.	**2**	**5**	**5**
*Duration, months (mean ± SD)*	12	26,4 ± 21,46	30,4 ± 22,19
**Conventional > Biological** ^*^ **therapy, n.**	**16**	**18**	**6**
*Duration, months (mean ± SD)*	33,8 ± 16,9	48,1 ± 26,55	46 ± 34,77
**Conventional > Biological** ^*^ **therapy + MTX, n.**	**2**	**4**	**0**
*Duration, months (mean ± SD)*	35,5 ± 4,94	90,75 ± 65,06	-

^*^Adalimumab, etanercept, infliximab, golimumab, ustekinumab.

**[#]** The treatment schedule was standardized, the doses were used as follows.

● MTX (7.5 vs15 mg/ week -per os or intramuscular injections).

● Cyclosporine (2.5 vs 5 mg/prokilo/die- per os).

● Adalimumab (40 mg every other week-subcutaneous injections).

● Etanercept (50 mg weekly- subcutaneous injections).

● Infliximab (5 mg/kg at 0, 2 and 6 weeks, then every 8 weeks-endovenous infusion).

● Golimumab (50 mg monthly-subcutaneus injections).

● Ustekinumab ( For patients weighing ≤100 kg (220 lbs), the recommended dose is 45 mg initially and 4 weeks later, followed by 45 mg every 12 weeks. For patients weighing >100 kg (220 lbs), the recommended dose is 90 mg initially and 4 weeks later, followed by 90 mg every 12 weeks. – subcutaneous injections).

The data in bold represent "macro-categories", while the data not in bold represent their "sub-categories".

### HCV infection

Overall, 15/224 (6.6%) patients had a positive serology for anti-HCV (11 males and 4 females, mean age 62 yrs). The main features of the 15 patients with HCV infection are reported in Table 1. Nine patients were affected by plaque-type psoriasis and 6 by psoriatic arthritis. None of them showed signs of cirrhosis. All HCV patients had baseline normal aminotransferase levels, 6/15 were HCV-RNA negative and 9/15 were positive (mean levels of HCV-RNA: 16.207.071 UI/ml, range 43600 – 515.000.000), and underwent immunosuppressive therapy. The schedules of treatment are reported in Table 2. Nine patients (60%) were treated with conventional drugs only (Metotrexate, Cyclosporine or a combination of the two drugs) for a mean time of 26 months and 6 patients (40%) were treated with conventional and biological drug, sequentially, for a mean time of 46 months. Serum liver functional tests in all cases showed no significant changes in AST or ALT levels and in viral load, from baseline to the last follow-up visit.

## Discussion

The reactivation of viral hepatitis, in particular HBV disease, has been widely reported in patients undergoing IT in different clinical conditions with particular frequency and severity in onco-haematological and transplant settings._Conversely, very few data are available in literature on the course of liver disease and the risk of HBV or HCV reactivation in patients with psoriasis receiving IT [19-25], the major part of them are case reports and series of patients.

Our study is the first observational analysis conducted in a larger cohort of psoriatic patients with a serological pattern of active or previous HBV or HCV infection, assessing the safety of different schedules of immunosuppressive therapy for a mean follow-up period of 48 months.

The main results of our investigation is represented by the absence of episodes of viral reactivation in HBsAg negative-HBcAb positive patients independently of the type of immunosuppressive (conventional or biological) drugs.

The risk of reactivation for the category of potential occult HBV infection is controversial and might be related to the clinical setting and type of IT. There are several reported cases of HBV reactivation in HBcAb positive patients with and without concomitant HBsAb positivity in patients who have undergone bone marrow transplantation or cytotoxic chemotherapy for lymphoma [31]. In these patients, the use of intense immunosuppression, monoclonal antibodies anti-lymphocyte B and T (anti-CD20) are particularly considered as risk factors. Conversely, in other clinical settings (gastroenterology, rheumatology and dermatology) the risk of reactivation in isolated HBcAb positive patients seems to be negligible or absent [20,22,32,33].

Our result seems to be in agreement with other previous reports limited by the observation of a small number of patients using biological agents for a short follow-up period. For example, the study of Prignano et al. [23] conducted in a cohort of 17 patients with psoriasis, using etanercept and/or adalimumab showed no significant changes in aminotransferase and in viral load levels during a follow-up period of only 6–8 months. Moreover, in the latest study by Navarro et al. [28], the safety of anti-TNF agents in 13 psoriatic HBsAg negative – HbcAb positive patients during a mean follow-up time of 28.6 months has been pointed out, suggesting a low risk of HBV reactivation in potential occult HBV infection.

Concerning patients with resolved HBV infection (HBsAg negative, HBcAb positive, HBsAb positive), there is only one reported case of viral reactivation in a dermatological setting during immunosuppressive therapy with ustekinumab, as described by Koskinas et al. [19]. In our study, no viral reactivation was observed in this category, confirming a low risk of viral reactivation in resolved HBV infection.

On the other hand, more data are available in literature for patients with chronic HBV infection (HBsAg positive). In total, 103 patients HBsAg positive with concomitant psoriasis and IT have been described in literature [20,24,25,34,35]; 56/103 of these patients were treated with concomitant antiviral therapy and only 5/56 (8.9%) showed HBV reactivation [35]. Differently, among the remaining patients without antiviral prophylaxis, 32/47 (68%) patients showed HBV reactivation during biological therapy [25,35]. We did not observe HBsAg positive patients in our series of patients; therefore, we can add nothing to what is already known in this category, which seems to be the only one at high risk of reactivation during biological therapies. In this perspective, the European guidelines for the use of anti-TNF therapy in psoriasis published in 2009 [36], the British Association of dermatologist guidelines for the use of biological interventions in psoriasis published in 2005 [37] and the Japanese guidance for use of biologics for psoriasis published in 2013 [38] consider active chronic hepatitis B as relative or absolute contraindication to the use of anti-TNF agents. Considering the success of biological IT in HBsAg patients using concomitant antiviral therapy, these guidelines seem far from clinical reality and current evidence. Only 5 cases of HBV reactivation in psoriatic patients treated with biological drugs (3 etanercept and 2 infliximab), using concomitant antiviral therapy (lamivudine) are reported in literature until now [35].

The prophylactic use of antiviral drugs is an important issue. Lamivudine prophylaxis has been suggested on the basis of its well demonstrated efficacy; however, other nucleotides/nucleosides should be preferred for their lower propensity to provoke drug resistance, especially if immunosuppressive therapy is scheduled for more than 12 months. The role of alternative anti-viral drugs, including entecavir and tenofovir, is still unknown and further studies are needed.

With regard to patients with HCV infection, the use of IT appears to induce lower frequency and severity of viral reactivation than in HBV. This finding is confirmed in our series of 15 HCV patients undergoing IT in which no viral reactivation was observed. Only one case of HCV reactivation during treatment with ustekinumab for psoriasis is reported in the literature [25].

## Conclusions

In conclusion, screening for HBV and HCV is now recommended for all patients with psoriasis, before starting all types of IT therapy. All patients with psoriasis should be tested for HBV (including HBsAg, HBcAb, HBsAb and HBV-DNA if needed) and HCV (including HCV-Ab and HCV-RNA if HCV-Ab positive) to assess the infection or vaccination status. HBV vaccination is mandatory in all seronegative patients. The current data that are available suggest that anti-TNFα and IL-12 and IL-23 blockers may also represent a therapeutic option in patients with concomitant HBV and/or HCV infection. The risk of HBV or HCV reactivation related to TNFα inhibitors seems to be low. The best strategy to adopt in HBsAg negative/HBcAb positive patients with or without HBsAb positivity and HCV positive patients would appear to be represented by periodic clinical and laboratoristic monitoring.

## Abbreviations

HBV: Hepatitis B virus
HCV: Hepatitis C virus
HBsAg: HB surface antigen
HBcAb: Hepatitis B core antibody
HBsAb: Hepatitis B surface antibody
IT: Immunosuppressive Therapy
TNF-a: Tumour Necrosis Factor-alpha
MTX: Methotrexate
OBI: Occult HBV Infection
ALT: Alanine Aminotransferase
AST: Aspartate Aminotransferase
GGT: Gamma-Glutamiltransferase
SD: Standard Deviation
Pso: Plaque-type Psoriasis
PsA: Psoriatic Arthritis
